# Supplementary material for: Targeting the actin nucleation promoting factor WASp provides a therapeutic approach for hematopoietic malignancies
Source: Nat Commun. 2021 Sep 22;12:5581. doi: 10.1038/s41467-021-25842-7 (PMC8458504; doi:10.1038/s41467-021-25842-7)
Supplement: Supplementary file 1 — Supplementary Information [file 41467_2021_25842_MOESM1_ESM.docx]

**Supplementary Information File:**

**Supplementary figure 1**


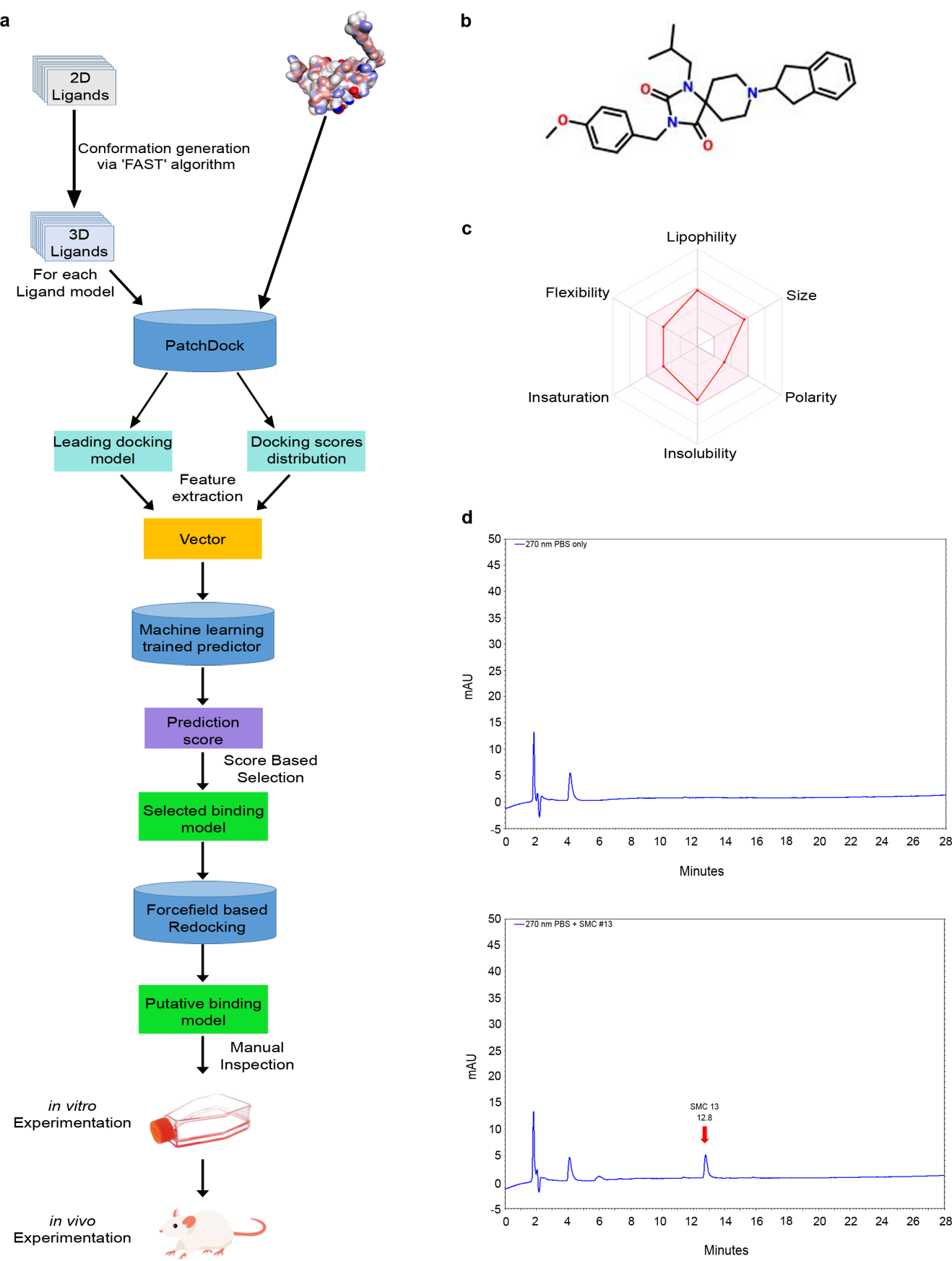


**Supplementary figure 1. Description of in-silico screening of SMC#13 and its characteristics. (a)** Detailed scheme of the in-silico workflow. **(b)** The structure of SMC #13:8-(2,3-dihydro-1H-inden-2-yl)-1-isobutyl-3-(4-methoxybenzyl)-1,3,8- triazaspiro[4.5]decane-2,4-dione. **(c)** SwissADME radar map showing pharmacological characteristics of SMC#13. **(d)** Distribution coefficient evaluation: Distribution coefficient (logD) was calculated by adding 1mg of SMC #13 to octanol-PBS (1:1) the PBS was used for the aqueous phase with a physiological pH of 7.4. The log D value was measured using HPLC and chromatograms were presented (n=3).

**Supplementary figure 2**


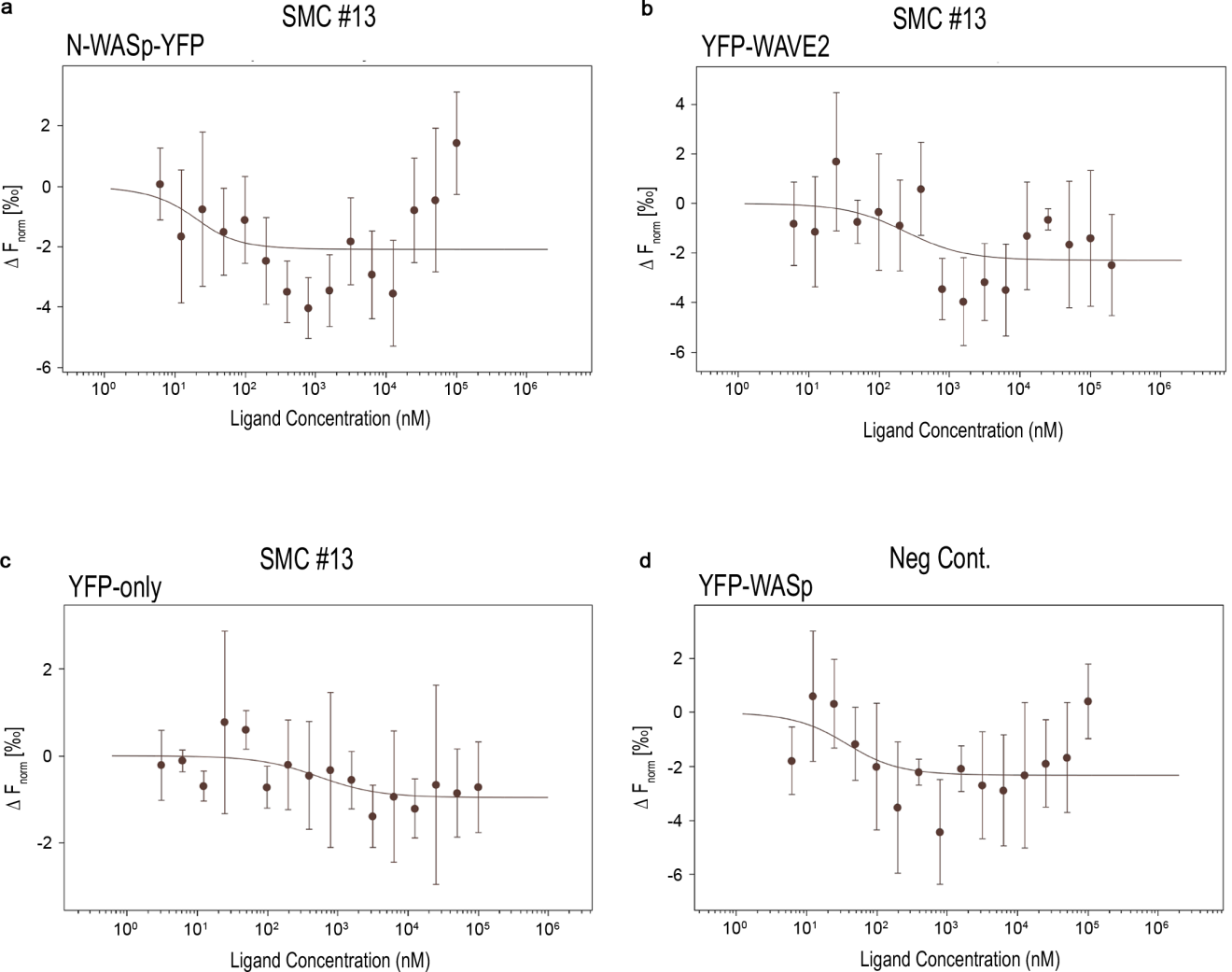


**Supplementary figure 2. SMC #13 does not bind WASp homologs.**

MST binding analysis between SMC #13 and **(a)** N-WASp, and **(b)** WAVE2. **(c)** HEK 293T cells expressing the YFP tag only were incubated with serially diluted (1.5 nM - 200 μM) SMC #13. The mixed lysates and SMC were then loaded into standard-treated Monolith^™^ capillaries, and the fluorescence of the samples was measured at 50% blue LED power in the Monolith NT.115 instrument at 60% MST power. Binding curves were generated by the NanoTemper Analysis 2.2.31 software, and the normalized fluorescence is plotted as a function of SMC concentration. Data shown as an average ±SD of n=8 (N-WASp), n=8 (WAVE2) n=4 (YFP tag only).. **(d)** MST binding analysis between irrelevant SMC Y-27632, a ROCK1 inhibitor (negative control), and YFP-WASp wt. Data shown as an average ±SD of n=6 (Y-27632).

**Supplementary figure 3**


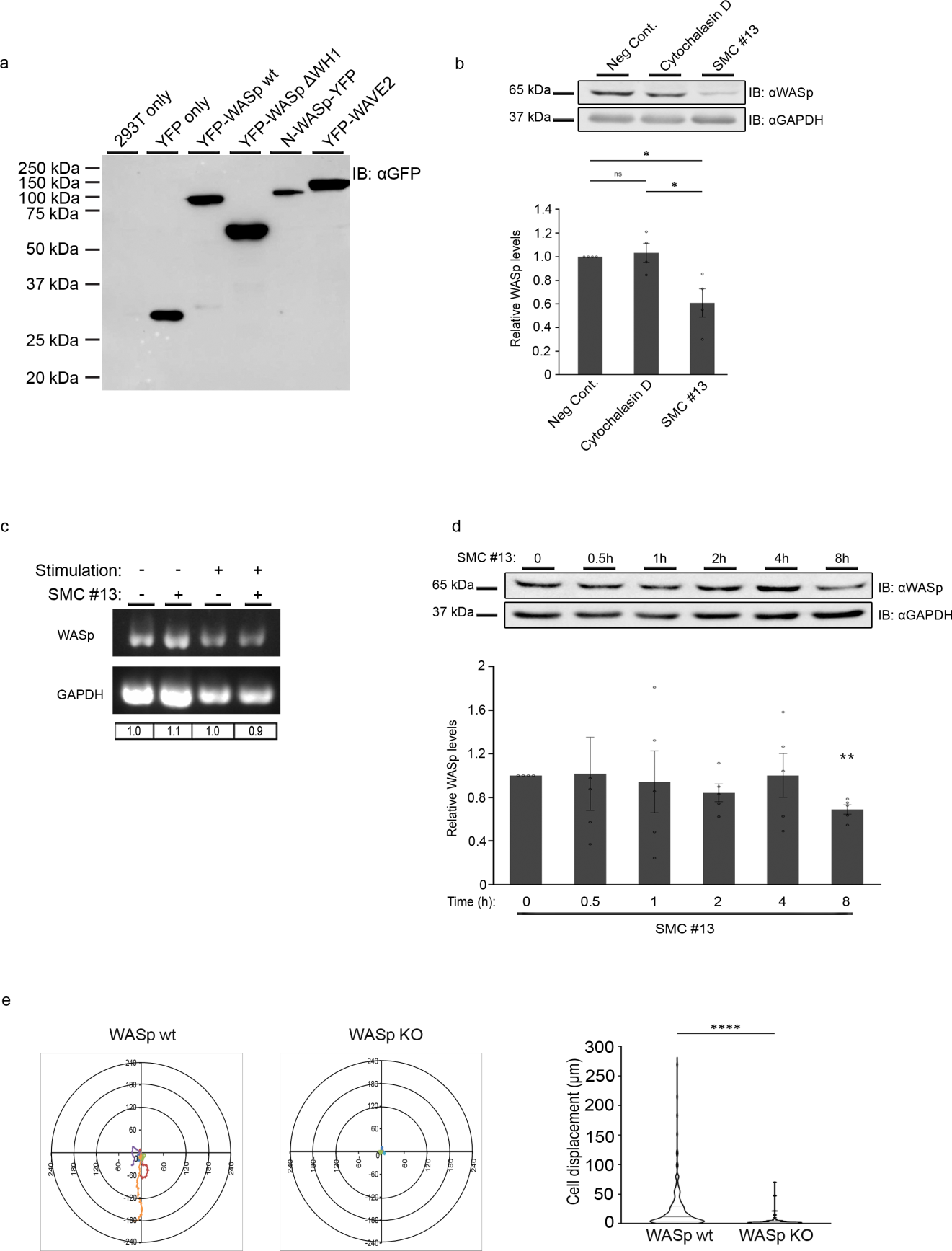


**Supplementary figure 3. Effect of SMC#13 on WASp RNA and protein levels. (a)** Western blot analysis of whole cell lysates from transiently transfected HEK293T cells expressing YFP-WASp wt, YFP-WAVE2, N-WASp-YFP**,** YFP-WASp ΔWH1, or YFP only plasmids were tested for quality control before MST or BLI**.** (**b**) PBMCs were incubated with 40 µM SMC #13 or vehicle for 24 h. Following incubation, the cells were co-stimulated with anti-CD3 (10 µg/ml) and anti-CD28 (10 µg/ml) and crosslinked with anti-mouse IgG (50 µg/ml) or left unstimulated. The RNA fraction was then extracted, and the amplified cDNA products were loaded onto an agarose gel. WASp was quantified relative to GAPDH as a loading control. Densitometric analyses of the bands were performed with ImageJ and are presented below for each lane (n=4; DMSO vs SMC#13, *P*=0.0226; Cytochalasin D vs SMC#13, *P*=0.0148). Values are shown as mean ± standard errors of the mean (SEM). The *P* values were calculated using one-way anova and Tukey’s multiple comparison test. N.S – not significant **(c)** Jurkat cells were incubated with either SMC#13 (40µM) or cytochalasin D (0.5μM). Cells were lysed and immunoblotted with anti-WASp antibody, and analyzed for WASp expression relative to GAPDH loading control. **(d)** Raji cells were treated with SMC#13 (40µM) for a 30 min period followed by media replacement without SMC #13 , and analyzed for WASp expression relative to GAPDH after the indicated times (0-8 h) (n=5, *P*=0.0056 DMSO vs 8h). Values are shown as mean ± SEM; the *P* value of a two-tailed Student’s t-test is shown. **(e)** YFP-WASp wt and WASp KO Jurkat cells were seeded on a pre-coated coverslip. Cell migration was assessed using Zeiss Observer Z1 inverted microscope. Left panel: cell tracking analysis of five cells from a representative movie of each group. Each line represents the pathway of a single cell. The graph on the right summarizes the mean cell displacement (*P*$\leq$0.0001) (WASp wt: n=179; WASp KO: n=88). Values are shown as mean ± SEM of three experiments; the *P* value of a two-tailed Student’s t-test is shown.

**Supplementary figure 4**


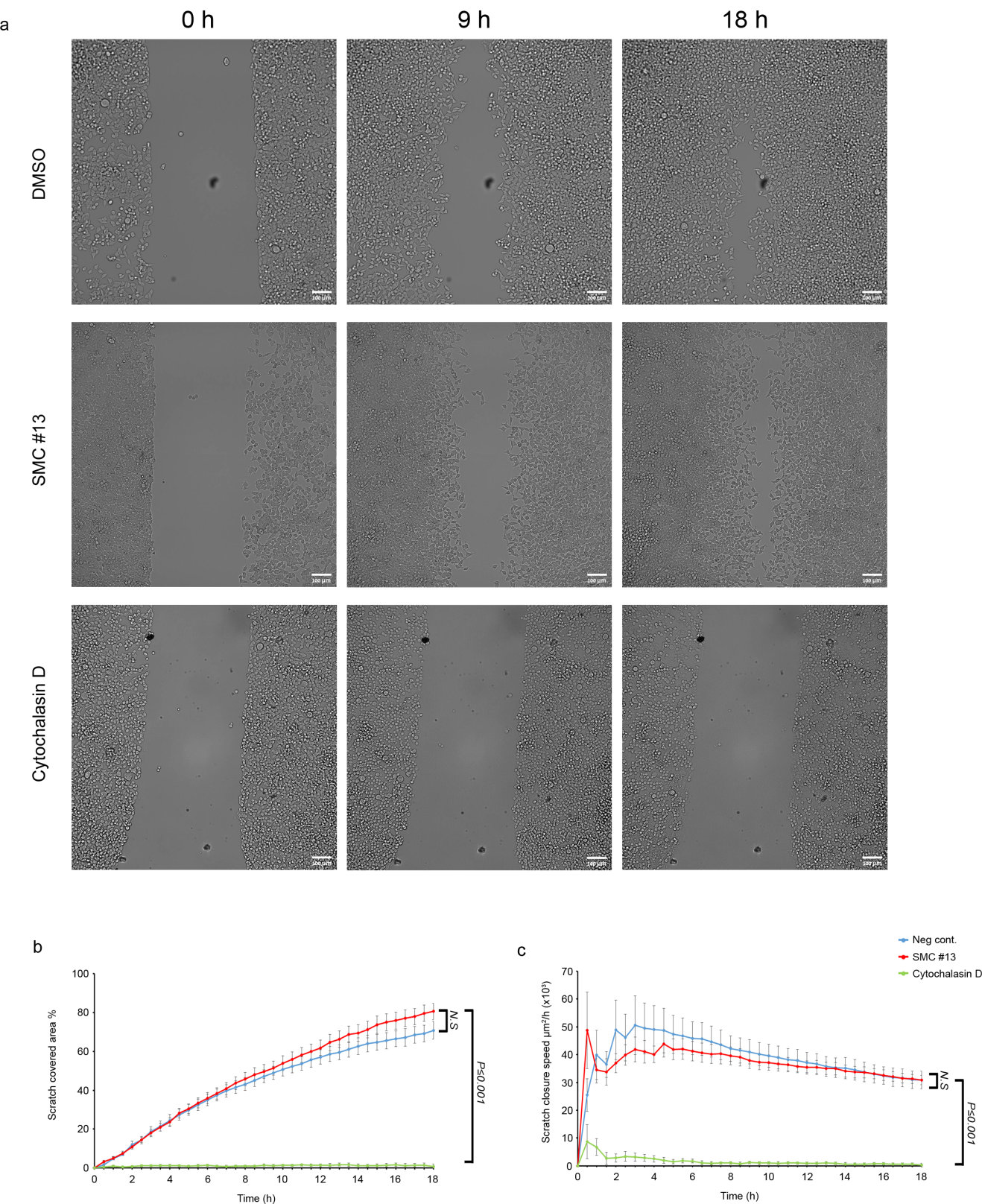


**Supplementary figure 4. Scratch-wound assay**

Mel1106 Melanoma cell line cells were incubated on culture dishes (as detailed in Materials and Methods) at 37°C and 5% CO_2_. Once cells reached high confluency, they were treated with 40 µM SMC #13, vehicle only, or with 0.5μM cytochalasin D as a positive control, scratches were introduced, and images were taken every 30 minutes. (**a**) Representative images at the indicated time points of 0, 9 and 18 hours (scale bar=100μm). (**b**) Percent scratch covered area (p$\leq$0.001). Values are shown as mean ± SEM of three experiments; the *P* value of a two-tailed Student’s t-test is shown. (**c**) Scratch closure speed µm^2^/h. 40μM SMC #13 (red), vehicle only (blue) or with 0.5μM cytochalasin D (green); (p$\leq$0.001). Values are shown as mean ± SEM of three independent experiments; the *P* value of a two-tailed Student’s t-test is shown.

**Supplementary figure 5**


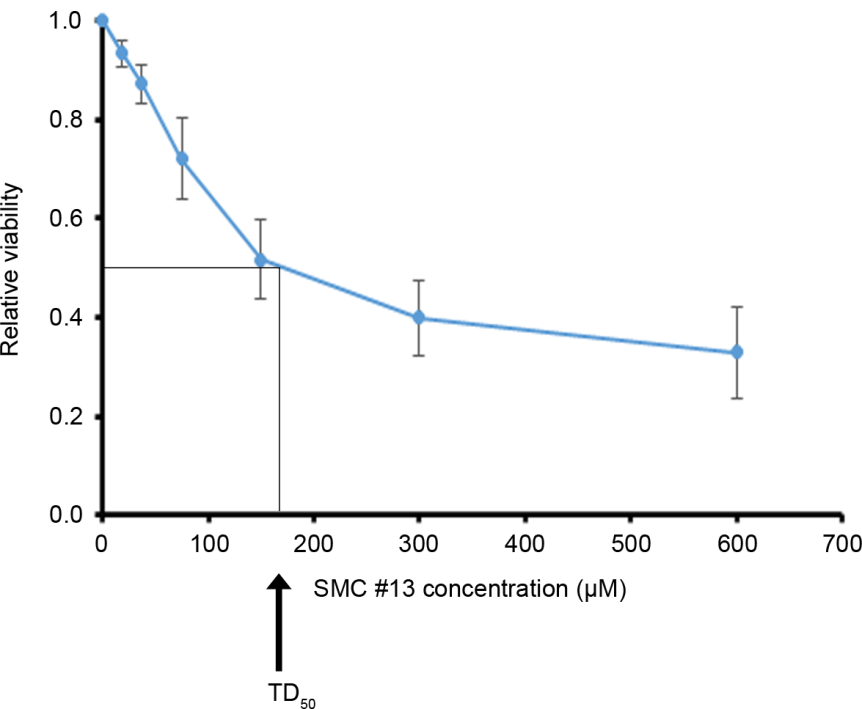


**Supplementary figure 5.** **Toxic dose 50 (TD50) of SMC #13.**

Cell viability following treatment with increasing concentrations of SMC #13 relative to cells treated with vehicle were performed using PI staining. Values are shown as mean ± SEM of three independent experiments.

**Supplementary figure 6**


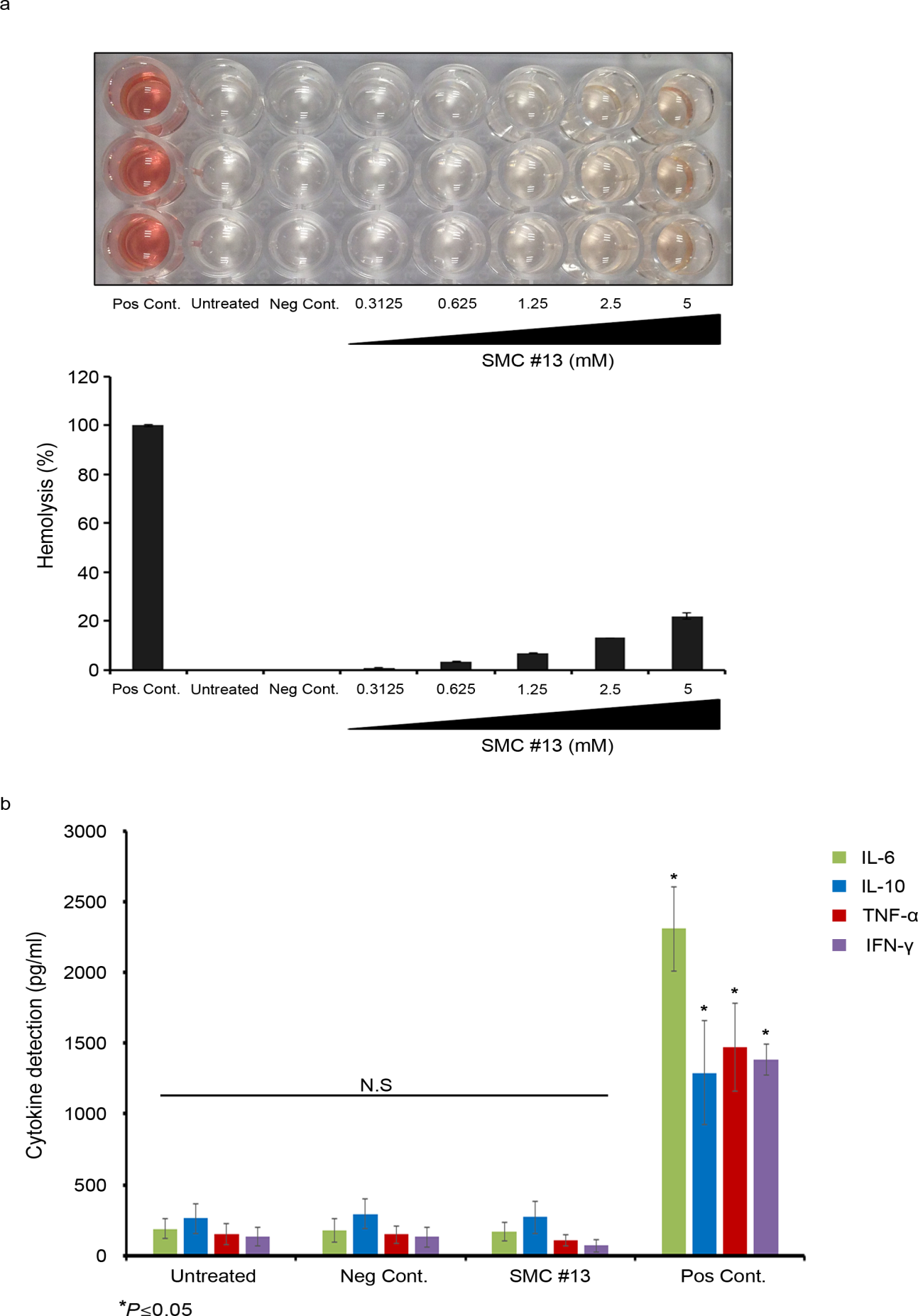


**Supplementary figure 6. Evaluation of *in-vitro* toxicity of SMC #13.**

**(a)** Hemolysis was tested following red blood cell incubation with the indicated concentrations of SMC #13 or vehicle for 3 h at 37˚C. Hemolysis was measured relative to cells incubated with water as 100% hemolysis (Pos Cont.) or untreated samples as 0% hemolysis. **(b)** PBMCs were incubated with 40 μM SMC #13, vehicle, or left untreated for 24 h. Positive control (Pos Cont.) cells were treated with PHA. Following incubation, supernatants from the samples were collected and examined for the indicated inflammatory cytokines by ELISA-based kit assay (SMC#13 vs Positive control; IL-6, *P*$\leq0.001;$IL-10, *P*=0.043; TNF-α, *P*=0.01; IFN-γ, *P*$\leq0.001$). Values are shown as mean ± SEM of three independent experiments; the *P* value of two-tailed Student’s t-test is indicated.

**Supplementary figure 7**


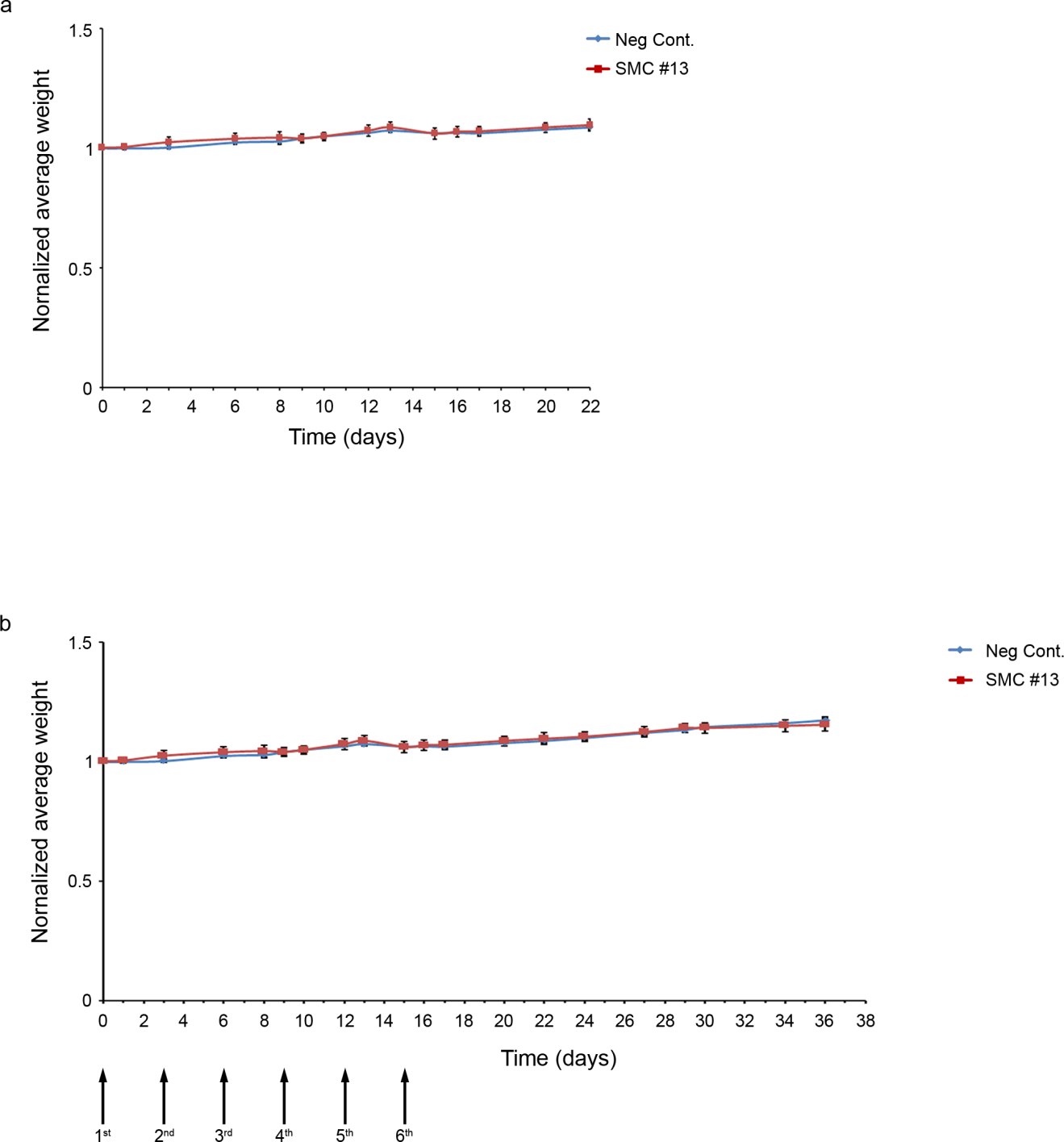


**Supplementary figure 7. Evaluation of *in-vivo* toxicity of SMC #13.**

**(a)** NOD/SCID mice were injected i.p. with a single dose of 100 mg/kg SMC#13 (n=12) or vehicle (n=10) and were weighed daily for 3 weeks. Error bars represent the SEM. **(b)** NOD/SCID mice were injected i.p. every 72 h with 100 mg/kg SMC#13 (n=8) or vehicle (n=9), for a total of six treatments. All mice were weighed daily. Values are shown as mean ± SEM of three independent experiments.

**Supplementary figure 8**


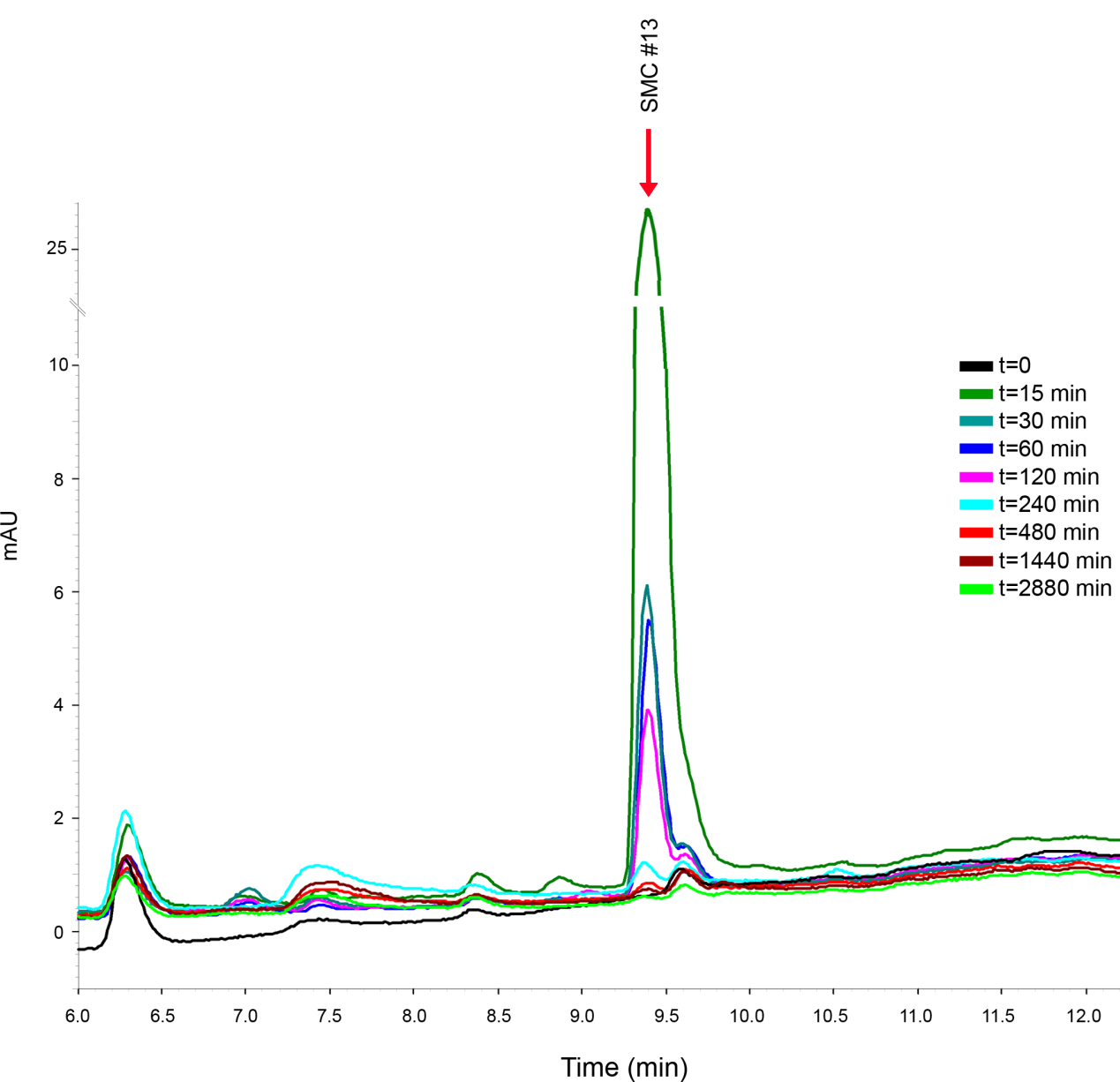


**Supplementary figure 8. HPLC analysis of SMC #13 following i.p. injection into SCID/NOD mice.**

NOD/SCID mice were injected i.p. with 100 mg/kg SMC #13. At the indicated time points, blood samples were obtained, plasma was extracted, and SMC#13 was detected by HPLC. Values are shown as mean ± SEM of three independent experiments.

**Supplementary figure 9**


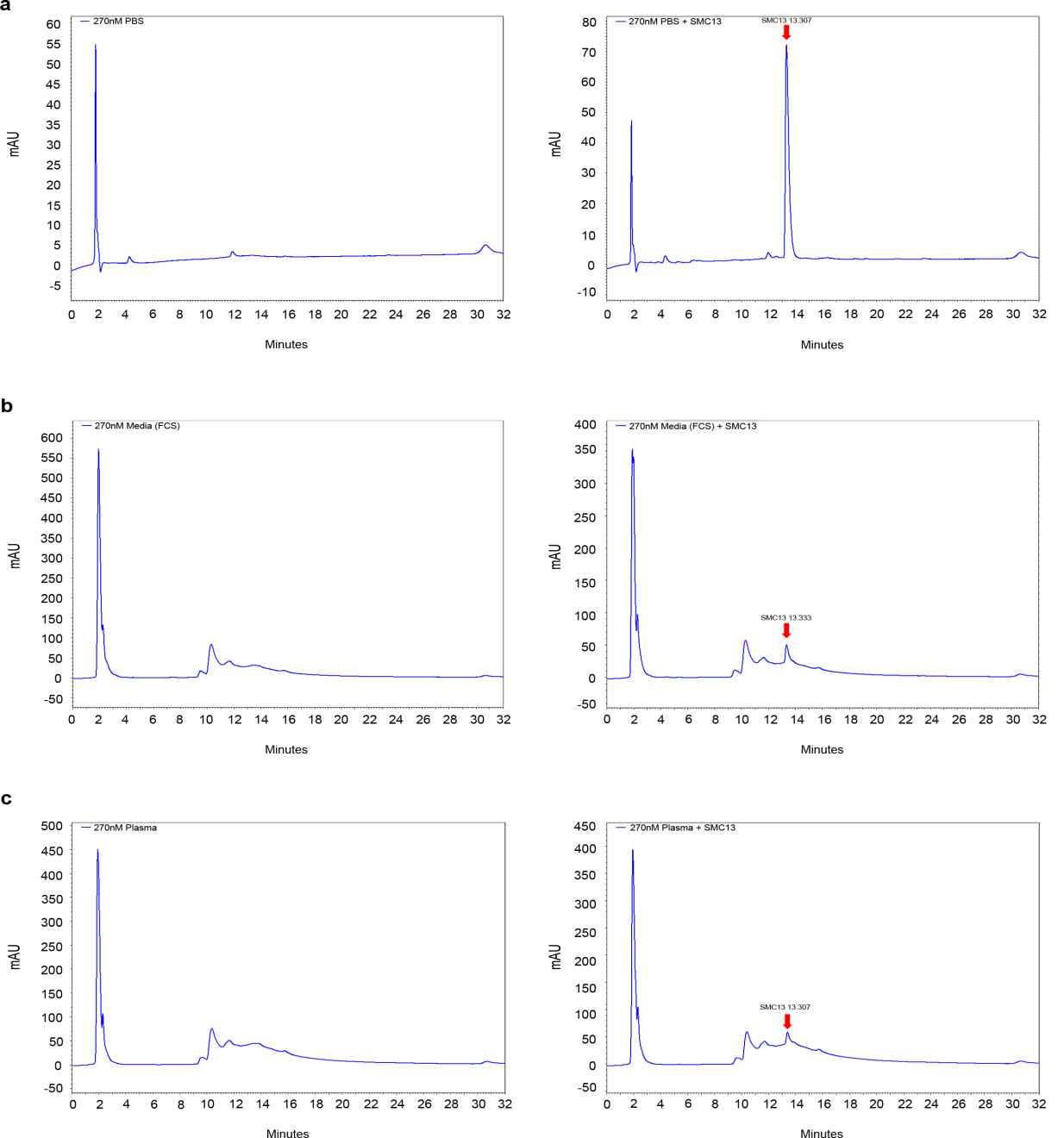


**Supplementary figure 9. Evaluating the plasma protein binding of SMC #13**

An equal concentration of SMC #13 (100μM) was incubated for 30 min with **(a)** PBS as a control, **(b)** complete medium supplemented with 10% serum or (C) full plasma. Unbound SMC was separated from the protein bound SMC #13 complex using a molecular weight cutoff of 10kD and the unbound SMC fraction was measured using HPLC. The flow through unbound fraction of SMC #13 was calculated relative to the initial concentration of 100μM.

**Supplementary figure 10**


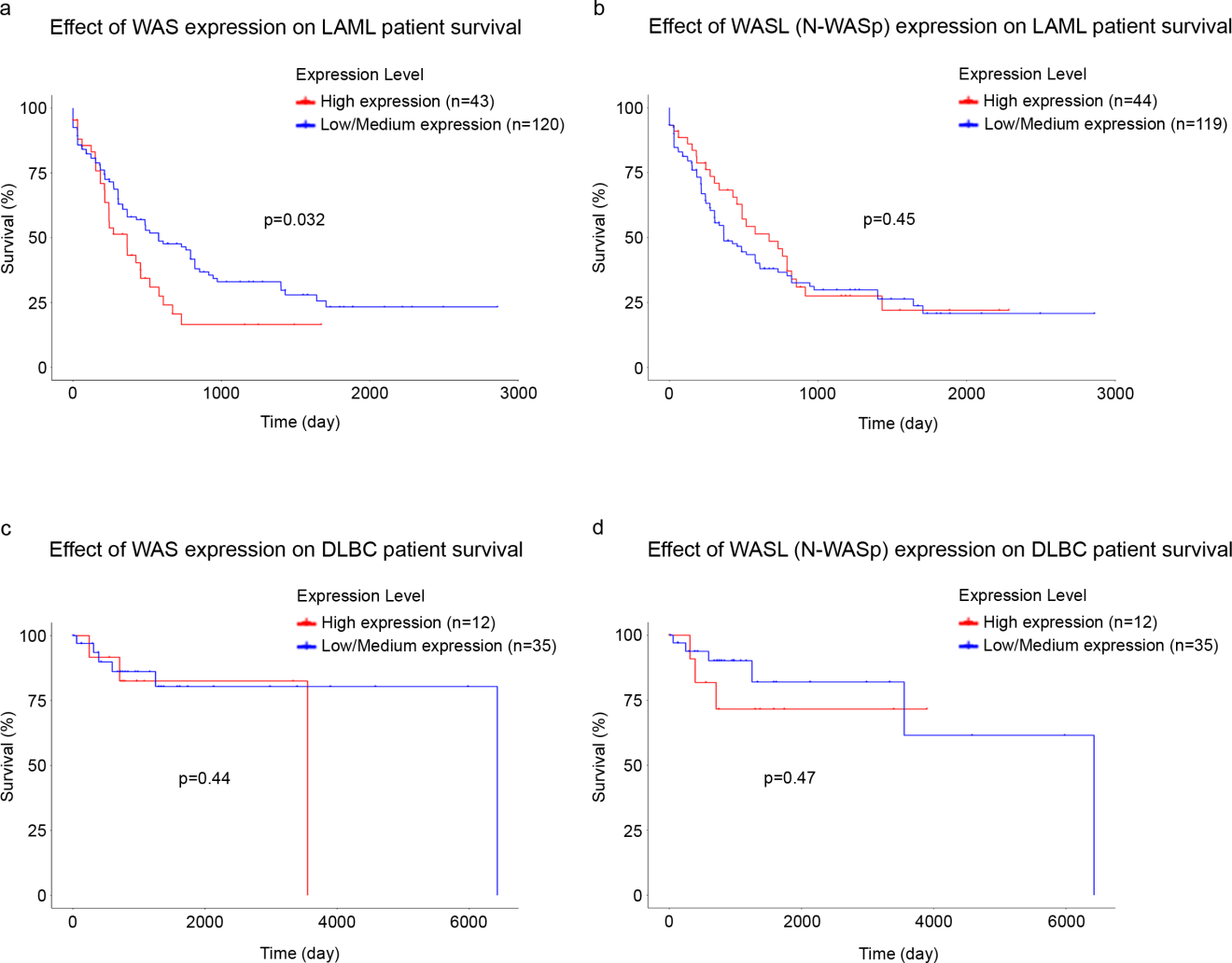


**Supplementary figure 10.** **Clinical survival curve of patients with AML and DLBCL relative to WASp and N-WASp expression.**

**(a**) Survival curve from TCGA data of 163 AML patients correlated to WASp expression. **(b)** Survival curve from TCGA data of 163 AML patients correlated to N-WASp expression. **(c)** Survival curve from TCGA data of 47 DLBCL patients in correlation to WASp expression. **(d)** Survival curve from TCGA data of 47 DLBCL patients in correlation to N-WASp expression. Raw data were obtained from the TCGA database and analyzed using the UALCAN portal: http://ualcan.path.uab.edu/index.html.The graphs are presented as Kaplan-Meier plots and the statistical analysis was performed using log rank test.

**Supplementary figure 11**

**For figure 2**


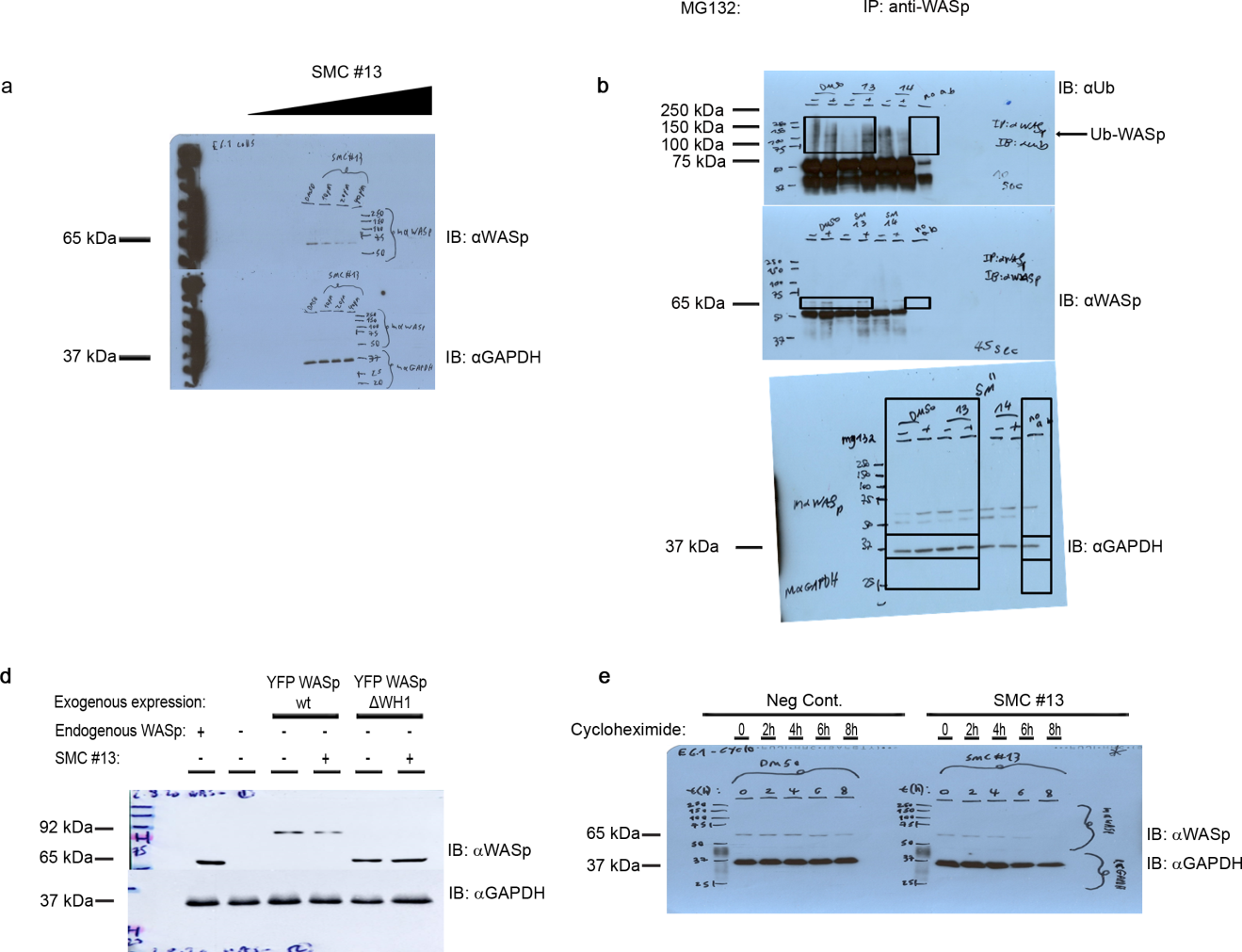


**For figure 3**


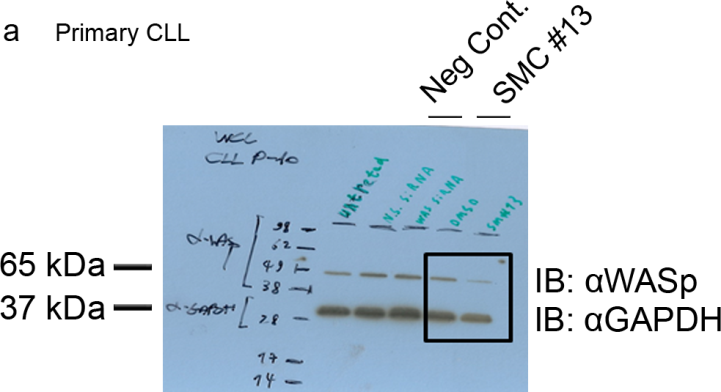


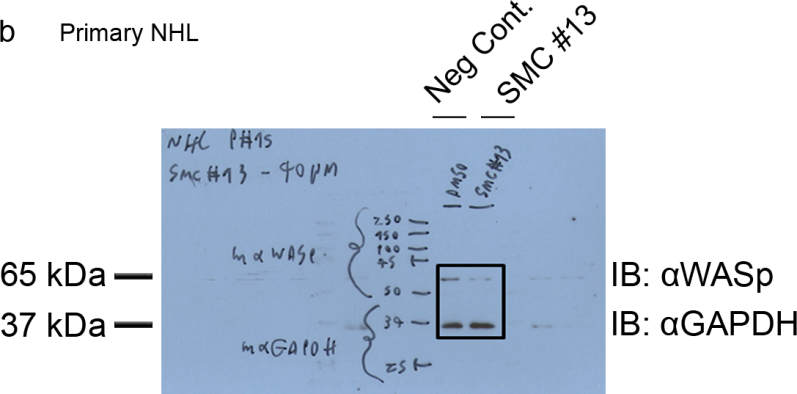


**For figure 4**


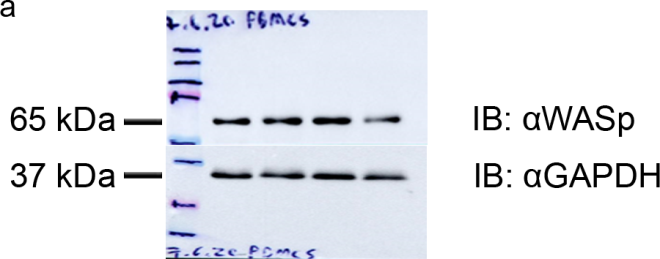


**For figure 6**


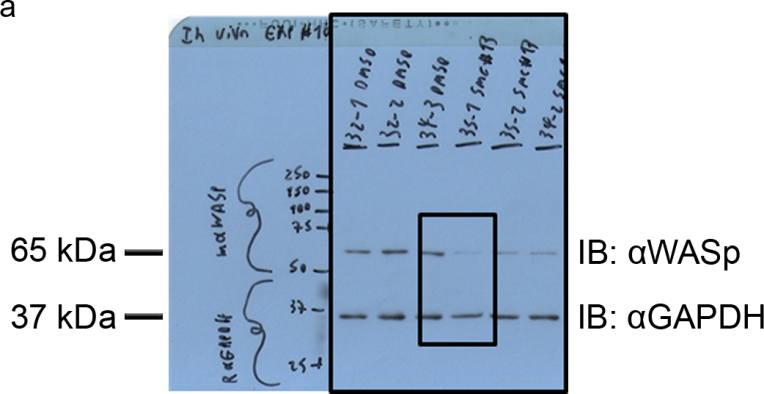


**For Supplementary figure 3**


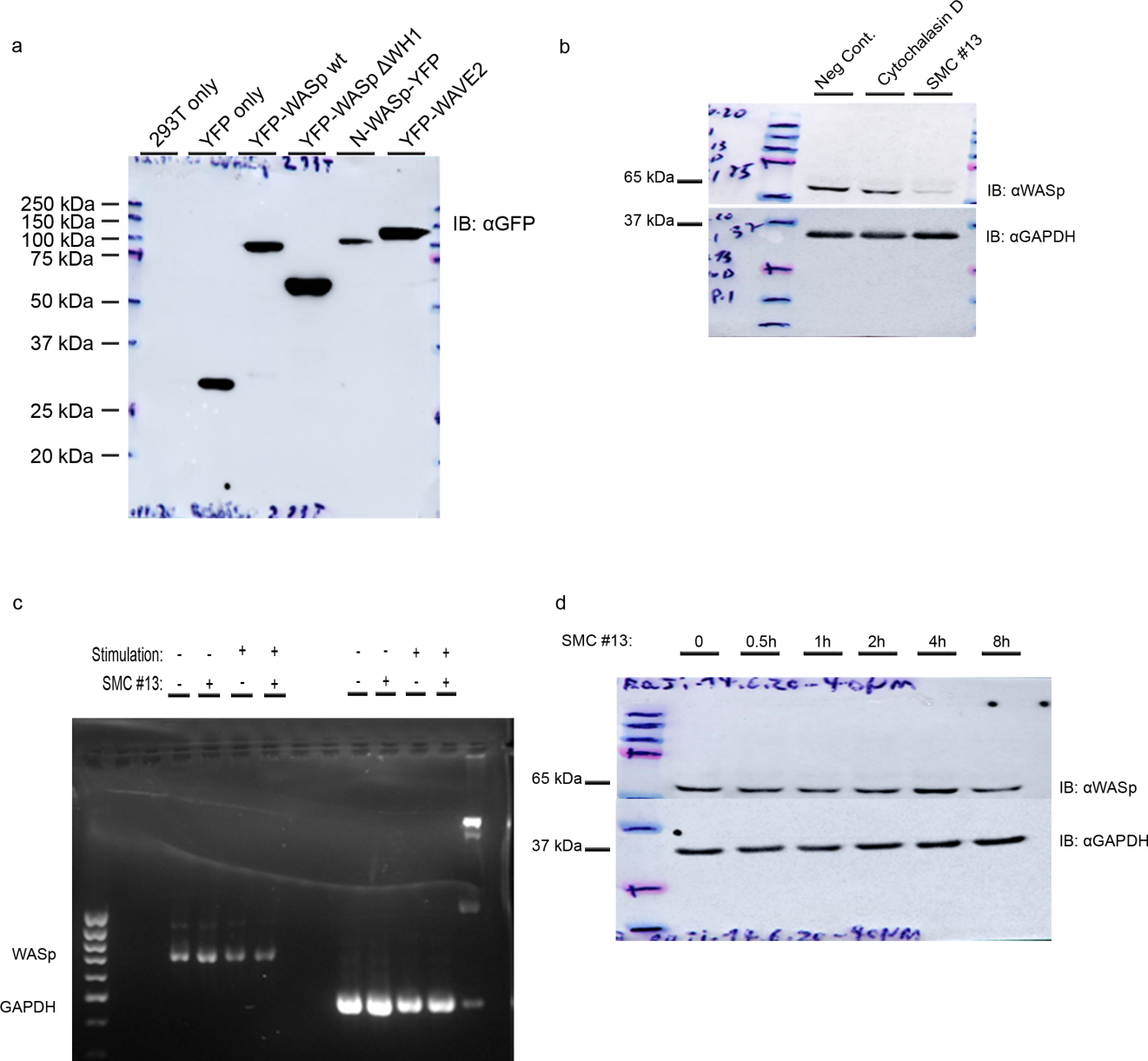


**Supplementary figure 11. Representative blots.**

The uncut and processed western blot images for Figure 2, 3, 4, 6 and Supplementary figure 3 are represented here.

**Supplementary figure 12**

**Figure 2g – Gating**

**Figure 6C - Gating**

**Supplementary figure 12. Flow cytometry gating strategy.** (a) The gating strategy of figure 2g (p<0.05, n=3) and (b) figure 6c (p<0.05, n=3) are described. The cells were characterized based on size and granularity/FSC-SSC plots. The relative mean fluorescence intensity is depicted as a histogram.

**Supplementary Figure 13a******

**Supplementary Figure 13b**

**Supplementary figure 13. Data of NMR and LS-MS of SMC#13.**

**(a)** Shows the LMS data of the compound SMC#13 and **(b)** Shows the NMR data for the compound SMC#13.
