# Supplementary material for: Targeting the actin nucleation promoting factor WASp provides a therapeutic approach for hematopoietic malignancies
Source: Nat Commun. 2021 Sep 22;12:5581. doi: 10.1038/s41467-021-25842-7 (PMC8458504; doi:10.1038/s41467-021-25842-7)
Supplement: Supplementary file 3 — Description of Additional Supplementary Files [file 41467_2021_25842_MOESM3_ESM.pdf]

Description of Additional Supplementary Files

Supplementary Movie 1. Migration of primary CLL cells pretreated with vehicle (negative control). Primary CLL cells, pretreated with the vehicle (DMSO), were plated over ICAM-1/SDF-1α-coated surfaces. The movie was prepared as indicated in Materials & Methods.

Supplementary Movie 2. Migration of primary CLL cells pretreated with SMC #13. Primary CLL cells, pretreated with 40 μM SMC #13, were plated over ICAM-1/SDF-1α-coated surfaces. The movie was prepared as indicated in Materials & Methods.

Supplementary Movie 3. Migration of primary NHL cells pretreated with vehicle (negative control). Primary NHL cells, pretreated with the vehicle (DMSO), were plated over ICAM-1/SDF-1α-coated surfaces. The movie was prepared as indicated in Materials & Methods.

Supplementary Movie 4. Migration of primary NHL cells pretreated with SMC #13. Primary NHL cells, pretreated with 40 μM SMC #13, were plated over ICAM-1/SDF-1α-coated surfaces. The movie was prepared as indicated in Materials & Methods.

Supplementary Movie 5. Migration of freshly isolated PBMCs from healthy donors pretreated with vehicle (negative control). Freshly isolated PBMCs from healthy donors, pretreated with the vehicle (DMSO), were plated over ICAM-1/SDF-1α-coated surfaces. The movie was prepared as indicated in Materials & Methods.

Supplementary Movie 6. Migration of freshly isolated PBMCs from healthy donors pretreated with SMC#13. Freshly isolated PBMCs from healthy donors pretreated with 40 μM SMC #13, were plated over ICAM-1/SDF-1α-coated surfaces. The movie was prepared as indicated in Materials & Methods.

Supplementary Movie 7. Migration of activated PBMCs from healthy donors pretreated with vehicle (negative control). Freshly isolated PBMCs from healthy donors were activated with PMA and ionomycin as described in Materials & Methods, pretreated with vehicle (DMSO), and plated over ICAM-1/SDF-1α-coated surfaces. The movie was prepared as indicated in Materials & Methods.

Supplementary Movie 8. Migration of activated PBMCs from healthy donors pretreated with SMC #13. Freshly isolated PBMCs from healthy donors were activated with PMA and ionomycin as described in Materials & Methods, treated with 40 μM SMC #13, and plated over ICAM-1/SDF-1α-coated surfaces. The movie was prepared as indicated in Materials & Methods.
