# Supplementary material for: Targeting the actin nucleation promoting factor WASp provides a therapeutic approach for hematopoietic malignancies
Source: Nat Commun. 2021 Sep 22;12:5581. doi: 10.1038/s41467-021-25842-7 (PMC8458504; doi:10.1038/s41467-021-25842-7)
Supplement: Supplementary file 12 — Reporting summary [file 41467_2021_25842_MOESM12_ESM.pdf]

nature portfolio

Corresponding author(s): Prof. Mira Barda-Saad
Last updated by author(s): Aug 23, 2021

Reporting Summary

Nature Portfolio wishes to improve the reproducibility of the work that we publish. This form provides structure for consistency and transparency in reporting. For further information on Nature Portfolio policies, see our Editorial Policies and the Editorial Policy Checklist.

Statistics

For all statistical analyses, confirm that the following items are present in the figure legend, table legend, main text, or Methods section.

n/a	Confirmed	
☐	☒	The exact sample size (n) for each experimental group/condition, given as a discrete number and unit of measurement
☐	☒	A statement on whether measurements were taken from distinct samples or whether the same sample was measured repeatedly
☐	☒	The statistical test(s) used AND whether they are one- or two-sided<br>*Only common tests should be described solely by name; describe more complex techniques in the Methods section.*
☒	☐	A description of all covariates tested
☐	☒	A description of any assumptions or corrections, such as tests of normality and adjustment for multiple comparisons
☐	☒	A full description of the statistical parameters including central tendency (e.g. means) or other basic estimates (e.g. regression coefficient) AND variation (e.g. standard deviation) or associated estimates of uncertainty (e.g. confidence intervals)
☐	☒	For null hypothesis testing, the test statistic (e.g. F, t, r) with confidence intervals, effect sizes, degrees of freedom and P value noted<br>*Give P values as exact values whenever suitable.*
☒	☐	For Bayesian analysis, information on the choice of priors and Markov chain Monte Carlo settings
☒	☐	For hierarchical and complex designs, identification of the appropriate level for tests and full reporting of outcomes
☒	☐	Estimates of effect sizes (e.g. Cohen's d, Pearson's r), indicating how they were calculated

Our web collection on statistics for biologists contains articles on many of the points above.

Software and code

Policy information about availability of computer code

Data collection	MO. Control for Monoloth NT.115, Synergy H1 - Gen5(3.08),<br>Flow cytometry: Kaluza acquisition software.<br>Microscopy: Zen software (v2.3), EZChrom v04, Zeiss LSM 510 META, LASX software for Leica SP8 confocal microscope (v3.0.14).<br>Bi-Layer Interferometry: Octet Red96 DataAcquisition-11_0_0_64d.
Data analysis	MO. Affinity Analysis V.2.1.2333, Data Analysis HT 11.0, IP lab software v4.0.<br>Kaluza and Flowjo analysis software (v9.7.6 and v10.3.0).<br>ImageJ 1.53c, ImageJ TrackMate plugin software v6.0.1, CellProfiler analysis software v3.0.<br>UALCAN analysis, Microsoft Office Excel v14.0, Graphpad v9.1.0 - Paired, unpaired t-test, one-way anova with post hoc multiple comparisons.

For manuscripts utilizing custom algorithms or software that are central to the research but not yet described in published literature, software must be made available to editors and reviewers. We strongly encourage code deposition in a community repository (e.g. GitHub). See the Nature Portfolio guidelines for submitting code & software for further information.

Data

Policy information about availability of data

All manuscripts must include a data availability statement. This statement should provide the following information, where applicable:

- Accession codes, unique identifiers, or web links for publicly available datasets
- A description of any restrictions on data availability
- For clinical datasets or third party data, please ensure that the statement adheres to our policy

Provide your data availability statement here.

Field-specific reporting

Please select the one below that is the best fit for your research. If you are not sure, read the appropriate sections before making your selection.

[x] Life sciences [ ] Behavioural & social sciences [ ] Ecological, evolutionary & environmental sciences

For a reference copy of the document with all sections, see nature.com/documents/nr-reporting-summary-flat.pdf

Life sciences study design

All studies must disclose on these points even when the disclosure is negative.

Sample size	No sample size was predetermined. For in-vitro assays, at least 3 independent experiments were preformed unless otherwise noted. For Imaging studies such as FRET, migration, IHC, and invasion assays, analyses were conducted on a large quantity of cells with the the number of cells, N, listed in the figure legends or in the text. For in-vivo experiments, between 4-6 mice were used per group.
Data exclusions	No data were excluded from final analyses.
Replication	All experiments were repeated with consistent results and n values are included in the figure legends.
Randomization	Randomization was used to divide up the animals for in vivo treatment study.
Blinding	Blinding is not relevant to this study since randomization was not applicable as the genetics were identical for all subjects in the same experiment. Mice were selected on the basis of genotypes and disease states.

Reporting for specific materials, systems and methods

We require information from authors about some types of materials, experimental systems and methods used in many studies. Here, indicate whether each material, system or method listed is relevant to your study. If you are not sure if a list item applies to your research, read the appropriate section before selecting a response.

Materials & experimental systems

n/a	Involved in the study	
[ ]	[x]	Antibodies
[ ]	[x]	Eukaryotic cell lines
[x]	[ ]	Palaeontology and archaeology
[ ]	[x]	Animals and other organisms
[ ]	[x]	Human research participants
[x]	[ ]	Clinical data
[x]	[ ]	Dual use research of concern

Methods

n/a	Involved in the study	
[x]	[ ]	ChIP-seq
[ ]	[x]	Flow cytometry
[x]	[ ]	MRI-based neuroimaging

Antibodies

Antibodies used	Anti-GFP (Roche, Clones 7.1 and 13.1) CAT#11814460001, 1:500 WB. Anti-WASp (Santa Cruz, Clone D-1) CAT# sc-5300, 1:1000 WB. Anti-GAPDH (Santa Cruz, clone FL-335) CAT# sc-25778, 1:1000 WB. Secondary Goat Anti-Mouse HRP (Jackson, polyclonal) CAT#115-035-003, 1:10,000 WB. Secondary Goat Anti-Rabbit HRP (Jackson, polyclonal) CAT#111-035-144 1:10,000 WB. Anti-LFA-1 (KIM127-non commercial hybridoma, ATCC- CRL2838), 1:1000 flow cytometry. Secondary Goat Anti-Mouse IgG1 (Jackson, polyclonal) CAT#115545-003, 1:2000 flow cytometry. Anti-Ubiquitin (Dako, polyclonal). CAT#z045801, 1:1,000 WB. Anti-Ki67 (Abcam, polyclonal) CAT#ab15580, 1:500 IHC. Anti-cleaved Caspase-3 (Cell Signaling, clone 5A1E). CAT#9664, 1:150 IHC, Alexa Fluor 568-conjugated goat anti-mouse (Invitrogen CAT# A21124) IHC 1:2000 , Alexa Fluor 594-conjugated goat anti-rabbit (Invitrogen CAT# A11012) IHC 1:2000, GFP-Trap (ChromoTek GFP-Trap® - gtb-250 121150) .

Validation	These commercially available antibodies validation data is provided on the manufacturer's website. Validation on the websites is conducted by comparing staining of isotype or control antibodies with the relevant targeting antibody. Data can be accessed through the following manufacturers websites: Roche (https://www.roche.com), Santa Cruz (https://www.scbt.com/home) Jackson (https://www.jacksonimmuno.com/), Dako (https://www.agilent.com/en/dako-products), Abcam (https://www.abcam.com), Cell Signaling (https://www.cellsignal.com), Invitrogen (https://www.thermofisher.com), ChromoTek (https://www.chromotek.com/)

Eukaryotic cell lines

Policy information about cell lines

Cell line source(s)	HEK293T, Raji, Jurkat E6.1 cell lines are from ATCC .
Authentication	Cell lines were not authenticated by genetic profiling.
Mycoplasma contamination	Cell lines were tested negative for mycoplasma contamination.
Commonly misidentified lines (See ICLAC register)	None

Animals and other organisms

Policy information about studies involving animals; ARRIVE guidelines recommended for reporting animal research

Laboratory animals	SCID/NOD and NRG male, 6-8 weeks mice were purchased from envigo or Jackson laboratories. All mice were housed in IVC caging, supplied with irradiated shredded corn cob bedding and irradiated mouse feed diet. The light-dark cycle was 12 hours. The ambient temperature of each room was set between 20 and 27C. The temperature inside the boxes generally sat between 22 and 24C. Humidity was set between 35 to 55%.
Wild animals	No wild animals used.
Field-collected samples	No field samples were collected
Ethics oversight	According to Bar-Ilan University ethics committee guildlines for animal welfare.

Note that full information on the approval of the study protocol must also be provided in the manuscript.

Human research participants

Policy information about studies involving human research participants

Population characteristics	NHL and CLL patient samples were obtained from recently untreated patients with informed consent in accordance with the declaration of Helsinki (0196-13-HMO) and approval by the Bar-Ilan University ethics committee, and in cooperation with Sheba medical center. Patient's personal characteristics including, age, gender, genotypic information, remained confidential according to Sheba Medical Center's policies. Blood samples from healthy donors were randomly collected and provided by Magen David Adom. No patient's identification information was provided.
Recruitment	Patients with diagnosed NHL or CLL were consented for blood samples and analyses.
Ethics oversight	The Bar-Ilan ethics committee in cooperation with Sheba medical center.

Note that full information on the approval of the study protocol must also be provided in the manuscript.

Flow Cytometry

Plots

Confirm that:

- [x] The axis labels state the marker and fluorochrome used (e.g. CD4-FITC).
- [x] The axis scales are clearly visible. Include numbers along axes only for bottom left plot of group (a 'group' is an analysis of identical markers).
- [x] All plots are contour plots with outliers or pseudocolor plots.
- [x] A numerical value for number of cells or percentage (with statistics) is provided.

Methodology

Sample preparation	For surface LFA-1 staining, cells were collected and washed twice with FACS buffer (PBS with 10% FBS-/-). Following incubation with primary anti-LFA-1 antibody for 30 minutes in 37 degrees Celsius, cells were washed twice with FACS buffer and stained with secondary anti-mouse IgG1 conjugated with Alexa Fluor 488 for 30 minutes on ice. Subsequently, cells were washed twice with FACS buffer and analyzed by flow cytometry.<br>For intracellular staining, cells were washed twice using FACS buffer. Cells were then fixed for 20 minutes in room temp using 3.7% PFA and subsequently permeabilized using 0.1% triton X-100 in PBS for 4 minutes in room temp. Cells were blocked for 45 minutes using PBS with 2% goat serum followed by staining with anti-WASp primary antibody for 45 minutes on ice. Cells were then washed twice with FACS buffer and stained with secondary anti-mouse IgG1 conjugated with Alexa Fluor 488 for 30 minutes on ice followed by FACS analysis.
Instrument	Gallios Beckman Coulter
Software	Collection software: Kaluza<br>Analysis software: Kaluza/ flowjo.
Cell population abundance	No sorting was performed.
Gating strategy	Gating was performed on a linear FSC/SSC plot. Consistent gating was performed on all samples and fluorescent analysis was preformed relative to control samples.

☒ Tick this box to confirm that a figure exemplifying the gating strategy is provided in the Supplementary Information.
